# Graduates’ perceptions of prosthetic and orthotic education and clinical practice in Tanzania and Malawi

**DOI:** 10.4102/ajod.v5i1.142

**Published:** 2016-06-10

**Authors:** Lina Magnusson, Harold G. Shangali, Gerd Ahlström

**Affiliations:** 1 Department of Health Sciences, Faculty of Medicine, Lund University, Sweden; 2 Department of Prosthetics and Orthotics, Faculty of Rehabilitation Medicine, Kilimanjaro Christian Medical University College, Tumaini University Makumira, United Republic of Tanzania

## Abstract

**Background:**

Maintaining and improving the quality of prosthetics and orthotics education at the Tanzania Training Centre for Orthopaedic Technologists is essential for the provision of appropriate prosthetics and orthotics services in African countries.

**Objectives:**

To describe how Tanzanian and Malawian graduates’ of the Diploma in Orthopaedic Technology perceive their education and how it could be improved or supplemented to facilitate clinical practice of graduates.

**Methods:**

Nineteen graduates from the diploma course in orthopaedic technology were interviewed and phenomenographic analysis was applied to the data.

**Results:**

Seven descriptive categories emerged, namely varied awareness of the profession before starting education, well-equipped teaching facilities, aspects lacking in the learning context, need for changes in the curriculum, enabling people to walk is motivating, obstacles in working conditions and the need for continuous professional development. All participants perceived possible improvements to the content and learning environment.

**Conclusions:**

Prosthetic and orthotic education can be better provided by modifying the content of the diploma programme by dedicating more time to the clinical management of different patient groups and applied biomechanics as well as reducing the programme content focusing on technical aspects of prosthetic and orthotic practice. Graduates were not prepared for the rural working conditions and the graduates desired continued training.

## Introduction

A tertiary education that provides good training conditions for high-quality rehabilitation service provision needs to be prioritised when it has been estimated that 0.5% of the population in developing countries require assistive devices such as orthotics and prosthetics (World Health Organization & International Society of Prosthetics and Orthotics 2005). National studies conducted in Malawi, Mozambique, Namibia, Zambia and Zimbabwe found that only 17% to 37% of individuals needing an assistive device received it. Gender inequalities where women had less access to assistive devices were observed in Malawi (women 14%, men 25%) (Loeb & Eide, 2004), and in Zambia (women 12%, men 16%) (Eide & Loeb, 2006). Orthotic and prosthetic devices and crutches facilitate or enhance user mobility (World Health Organization & United States Agency for International Development 2011). Despite efforts made by international, national and local stakeholders, mobility needs are not being met (World Health Organization & United States Agency for International Development 2011). In order to provide prosthetic and orthotic services, educated staff is needed.

Two surveys by the United Nations (South-North Centre for Dialogue and Development 2006) and the World Health Organization (World Health Organization 2004) assessed the global situation in light of the standard rules on the equalisation of opportunities for persons with disabilities and level of implementation by states in 2004. About half of the African countries that responded had not supplied people with disabilities with assistive devices including prosthetics and orthotics (South-North Centre for Dialogue and Development 2006). All countries reported that they could not provide services for everyone or provide maintenance and repair services and that services for people in rural areas were lacking. Assistive devices were free to users in a few African countries through government provision or external donors, whilst in other countries users were asked to pay based on income (World Health Organization 2004). Prosthetic and orthotic services need to be provided in low-income countries to address the Convention of Rights for Persons with Disabilities (CRPD), which dictates access to rehabilitation services (Article 26) and personal mobility (Article 20) (United Nations 2007).

Africa has five prosthetic and orthotic tertiary education programmes that are recognised by the International Society of Prosthetics and Orthotics (ISPO): the Tanzania Training Centre for Orthopaedic Technologists (TATCOT); the Ecole Nationale des Auxiliaires Médicaux, in Togo; the Sudanese Diploma in Prosthetics and Orthotics; and the Orthopaedic Technique Vocational and Educational Training Programme in Ethiopia and the University of Rwanda (International Society of Prosthetics and Orthotics 2015). There are also education programmes not yet recognised by the ISPO in South Africa, Kenya, Uganda and Morocco, and the International Committee of the Red Cross (ICRC) provides module courses in Ethiopia. Trained personnel within the field of rehabilitation, including prosthetists and orthotists, are especially lacking in Africa (Raab 1992; Shangali 2002). It is important that the education offered is of high-quality (World Health Organization & International Society of Prosthetics and Orthotics, 2005) as the trained prosthetic and orthotic professionals often become managers and trainers involved in the in-service training of those who are providing the required services. It is necessary to determine if the education currently provided meets the needs of people with disabilities requiring prosthetic and orthotic services in Africa.

TATCOT was founded 1981 with the objective of training prosthetist/orthotist students from a number of countries in English-speaking Africa (Shangali 2002). The school was developed in accordance with, and continues to follow, the international standards whereby the curriculum divides prosthetic and orthotic education into Categories I, II and III (World Health Organization & International Society of Prosthetics and Orthotics 2005). The impact report of the training at TATCOT for prosthetic and orthotic personnel in relation to the ISPO Categories I and II standards revealed that the effect on the establishment of services, delivery of appropriate prosthetics services, clinical leadership and professional communities was satisfactory in Tanzania, Kenya and Uganda. However, emphasis should be laid on the implementation of a sound national referral system in order to improve client assessment, develop local solutions and promote quality standards (Sexton, Shangali & Munissi 2012). It was reported that only a few people with disabilities had access to rehabilitation services in Tanzania and Malawi (South-North Centre for Dialogue and Development 2006). The existing prosthetic and orthotic services in Tanzania and Malawi were free to some users through government provision or external donors, whilst other users needed to cover part or all of the costs themselves. The outcome of this study will present the Tanzanian and Malawian professionals’ perspective on how prosthetic and orthotic graduates can be better prepared for the regional conditions and needs.

The aim of the study is to explore how Tanzanian and Malawian graduates’ of the Diploma in Orthopaedic Technology perceive their education and how it could be improved or supplemented to facilitate clinical practice of graduates.

### Contribution to the field

The study contributes to description of critical areas that need to be considered in the forthcoming review and update of the international standards and guidelines for training personnel in Africa, Asia and South America for prosthetics and orthotics services (World Health Organization & International Society of Prosthetics and Orthotics 2005). The results of this study can also contribute to the local curriculum review at TATCOT.

## Method

### Design

A qualitative phenomenographic approach with individual semi-structured interviews was used. Phenomenography (Marton 1981; Reed 2006) focuses on participants’ perceptions and description of phenomena. It is a qualitative method developed within pedagogic research. The focus in phenomenography concerns how something appears to the participants and their subjective perceptions is collected through semi-structured interviews (Linder & Marshall 2003; Marton 1981). Data collection methods typically include interviews with a purposive sample of participants, with the researcher working towards an articulation of the interviewee’s varied perceptions that are as complete as possible (Reed 2006). Data are collected to capture a pre-reflective level of consciousness (Marton 1981; Reed 2006), and the result is a description on a collective level based on conceptions obtained from individual interviews. Description is important because our knowledge of the world and of the qualitative similarities and differences varies between different people. A phenomenographic data analysis sorts perceptions which emerge from the data collected into specific conceptions which generate categories of description (Marton 1986). Phenomenography does not involve interpretation of underlying meaning such as many other qualitative methods and does have the aim to describe the variation in the data (Marton 1986; Reed 2006).

### Sample and participants

Between 1996 and 2009, 131 students from 20 African countries graduated from the Diploma in Orthopaedic Technology course of education at TATCOT according to the curriculum designed in 1996. The 3-year education programme in prosthetics and orthotics was taught in English. The majority of graduates returned to their home country after graduation. To obtain variation in perceptions, a strategic sample of these graduates was drawn that accounted for variation in sex and employment situations. Names and details of workplaces of graduates were provided by TATCOT. The sample was drawn from a population comprising graduates from Tanzania (*n* = 35) and Malawi (*n* = 7). Nineteen graduates of those (*n* = 42) who completed the Diploma in Orthopaedic Technology course at TATCOT were selected. The sample comprised a third of the, eligible graduates from Tanzania (*n* = 12) and all graduates from Malawi meeting the inclusion criteria (*n* = 7). None of the graduates asked to participate in the interviews declined participation. Four women and 15 men, with an average age of 34 years (range of 27–47 years) were interviewed. The graduates had an average of 5 years’ experience (0.5–13 years) and were able to communicate well in English. The employment situations of graduates in Tanzania and Malawi were government-run workshops (*n* = 9), non-governmental organisations (*n* = 4), private practice (*n* = 1), teaching institutions (*n* = 3) and further education by studying for a BSc in prosthetics and orthotics (*n* = 2). In Tanzania, both urban and rural employment situations were represented. In Malawi, all the graduates worked in urban areas.

### Interview and procedure

Data were collected in Tanzania and Malawi during 2009–2010. Nine interviews were conducted by the first author LM and 10 interviews by two other Swedish prosthetists/orthotists. The interviews were carried out face to face, started with background questions and demographic questions followed by two major open questions. *How do you perceive your education at TATCOT? How do you perceive your education in relation to your daily clinical practice?* Furthermore, predetermined probing follow-up questions were asked to complement the main questions and covered areas related to prosthetic orthotic education, entering the workforce, professional development, how they handled the situation as new graduates and what knowledge they lacked and how the TATCOT education had influenced their performance. The predetermined follow-up questions were assessed as necessary to keep the interview sessions similar because the interviews were conducted by three different interviewers.

The interview guide was used in a previous study conducted in Pakistan (Magnusson & Ramstrand 2009). Before beginning, a pilot interview was conducted and minor adjustments to the content of the questions were made. The researcher, L.M., trained the other interviewers to ensure that interviews were carried out consistently. Briefing sessions were conducted after the three first interviews, where the interview situations were discussed and sound files were listened to together in order to strive for consistency in the use of the interview guide and probing and follow-up questions. Recorded interview sessions lasted 20–60 minutes. The 19 interviews were transcribed verbatim and the sound files were listened to, to verify correct transcription and have a sense of the overall interviews.

### Data analysis

The phenomenographic analysis was applied in four phases (Marton 1981; Reed 2006). First, statements in the transcripts relating to the aim were identified. Second, 442 statements of the participants’ perceptions were abstracted into conceptions. Third, similarities and differences in conceptions were analysed and grouped into descriptive categories and are shown in Table 1 (Marton 1996). Fourth, the analysis focused on the relationships between the conceptions within each descriptive category and how the descriptive categories related to each other. Data analysis was conducted by authors LM and GA.

**TABLE 1:** Example of data analysis process.

Statements	Participants’ perceptions	Conception	Descriptive category
‘This is something bad. Some families, when they get a kid who is disabled, the Maasai used to leave them in the house, just hide them, and then you find a kid who has been hiding and now he is no longer a kid. He might be 22 or 23, and he has been in the house for all of his years. He is psychologically defected, and you bring him out and he is just wondering, what is this now, and you see a lot of contractures.’ (Interviewee 11, Tanzania)	Met disabled patients who had been hidden all their lives by their families and are now adults with psychological defects.	Working in rural conditions had different demands	Obstacles in working conditions
‘Now we have problem with diabetic patients. We have a lot of amputation from ulcers, there also the way that we are fabricating the trans-tibial prosthesis. There are problems with volume and wounds patients suffer from when they exercise, walking and then they get a wound again. So maybe there is a way we could make it fit better to the stump, so we can learn to make a proper socket with a fit. That is why I ask one of my teachers, just how to make something that is proper, and he says the most important thing is to take measurements everywhere so that the fitting should be exact. Those are the things I would like to learn more about.’ (Interviewee 14, Tanzania)	Diabetes is an increasing diagnoses causing amputations. There might be a better way we can learn to make prosthesis for diabetic patients to avoid ulcers.	Advanced prosthetic technology need to be included in education	Need for changes in the curriculum

## Results

Seven descriptive categories illuminate the variation in how education and clinical practice were conceptualised and understood by TATCOT graduates (Table 2). The descriptive categories and conceptions are presented below and each conception is exemplified with a quote.

**TABLE 2:** Graduate perspectives of the prosthetic and orthotic education curricula and profession.

Descriptive categories Conceptions	Number of statements	Number of interviews
**Varied awareness about profession before starting education** Low awareness of profession Desire to work with persons with disabilities	13 5	2–8, 10–11, 13, 15 5, 7, 17–19
**Well-equipped teaching facilities** Good workshop facilities Books were available at library, but insufficient Satisfaction with hostel facilities	19 18 16	2–19 2–17, 19 1, 3–4, 6–8, 10–11, 13–19
**Aspects lacking in the learning context** Adequate teaching but can be improved Desire for teachers with higher degrees Inequality in treatment of students	28 10 36	1–6, 8–15, 18–19 4, 11, 13–15, 17 1–6, 8–16, 19
**Need for changes in the curriculum** Need for the addition of advanced prosthetic and orthotic technology Drop technical drawing, metalwork, woodwork	64 26	1–7, 9–19 1, 3–4, 9, 11–12, 14–19
**Enabling people to walk is motivating** Helping people with disabilities is motivating Low-status profession	29 27	2–19 1–4, 6–9, 13–15, 18, 19
**Obstacles in working conditions** Varied support from senior staff and other professionals Lack of materials Different demands when working in underserviced and less resourced settings	45 17 34	1–2, 4–13, 14–19 3, 5–6, 8, 11–14, 17–19 3, 8, 10–12, 15, 17, 19
**Need for continuous professional development** Desire for continued training Suggested ways of keeping updated	40 15	1, 4–9, 11–17 1, 3, 6–7, 11

### Varied awareness about profession before starting education

#### Low awareness of profession

Participants perceived they had very limited knowledge about the prosthetic and orthotic professions before starting their education. The provision of information about the course and profession before they started their education was perceived as a required improvement. Participants stated that they enrolled in the prosthetic and orthotic education programme because they saw it as an opportunity for an education, rather than because they were choosing to become a prosthetist/orthotist.

‘I didn’t know what prosthetics and orthotics was before I started learning all about it in TATCOT.’ (Interviewee 5, Malawi)

### Desire to work with people with disabilities

Participants perceived that the profession was attractive because they wanted to improve the lives of people with physical disabilities. The desire to help was sometimes described as having originated from a relative in need of assistive devices or being influenced by a relative working in health care. Participants described how they became aware of the profession and the education programme through a school visit and from medical professionals and people using assistive devices.

‘I did not know about prosthetics and orthotics, but I saw some people with [*a missing limb*] and I asked them and they said this limb is fabricated, and I asked, where did you get that? Most of them answer that TATCOT fabricate these things.’ (Interviewee 18, Tanzania)

### Well-equipped teaching facilities

#### Good workshop facilities

Participants perceived the classrooms as good, and the workshop facilities were described as having the necessary machines and tools. The facilities were perceived as noisy, but ear protection was available.

‘The workshop is good and fully equipped. I don’t think they are missing any machines.’ (Interviewee 18, Tanzania)

#### Books were available at library, but insufficient

Participants perceived good availability of medical books through the medical library, but limited access to books about prosthetic and orthotic technology. These books were also considered very expensive to buy. The resource centre was perceived as often closed and the Internet as slow. A need to introduce scientific journals was described.

‘For other subjects it was okay as it is a big institution with a big library but for prosthetics and orthotics it was a problem, but for medical book there was no problem and we could share books.’ (Interviewee 6, Malawi)

#### Satisfaction with hostel facilities

Participants enjoyed the hostel facilities and described the environment as nice and peaceful and the accommodation as excellent. Participants emphasised the possibilities to cook, the access to the refrigerator and the television in the hostel.

‘I was very happy at the hostel. I would like to tell you within this environment, that TATCOT hostel is better than other hostels.’ (Interviewee 1, Tanzania)

### Aspects lacking in the learning context

#### Adequate teaching but can be improved

Participants had good perceptions of practical classes with teachers, in which they perceived they had ample opportunities to meet different patients and ask questions. However, the participants also perceived there was room for improvement in the teaching methodology. Specifically, more problem-solving teaching methodology was desired. The participants would have liked more clinical training and more adequate examples presented in lectures. They described that some teachers lectured by reading aloud from a book, which they perceived as very poor teaching. Possible improvements suggested included the inclusion of demonstrations in the subject, presentations and the assignments related to the clinical aspects of the field.

‘I think you have to lead the students to discover things themselves, while in TATCOT you taught saying ‘this is like this you can’t use any other way’, so you have to do it the way they taught you. If you do it another way, your marks will get reduced, so the best way would have been to give the problem to the students and they should try and solve it and come up with something, and in that way they will never forget.’ (Interviewee 9, Malawi)

#### Desire for teachers with higher degrees

The participants perceived a need to improve the competence of the teachers. The participants desired teachers with a degree higher than a diploma from TATCOT in prosthetics and orthotics. Participants described that many good teachers have left TATCOT. Newly graduated teachers were perceived as often not having their own teaching material and being unsure in the knowledge they taught. It was viewed that teachers should preferably be Tanzanians and be further educated, having completed the bachelor programme in Tanzania or internationally, outside Africa.

‘I think it is lacking in qualified teachers, many teachers have gone away. Let’s get those graduates that are getting from the diploma on a higher level so they can become teachers.’ (Interviewee 13, Tanzania)

#### Inequality in treatment of students

Participants perceived both equal and unequal treatment by teachers and staff during their education. The participants described the education programme as having mainly male students. Women and Tanzanian students were perceived as receiving better treatment than international male students. Participants also described how teachers treat students in non-respectful ways, and such behaviour needed to change.

‘They would show that they like the local Tanzanian students more than the others [*students from other African countries*].’ (Interviewee 6, Malawi)

### Need for changes in the curriculum

#### Need for the addition of advanced prosthetic and orthotic technology

The participants perceived a need to modify the curriculum by dedicating more time to the clinical management of different orthotic and prosthetic patient groups and other subjects such as applied biomechanics (Table 3).

**TABLE 3:** Need for changes in the curriculum and need for professional development.

Variables	Additional curricula statements (*n*)	Continued training statements (*n*)	Remove from curricula statements (*n*)
**Items suggested as additions or topics to be covered in more detail in curricula and in desired continued training**			
**Orthotic technology**			
Spinal orthotics	10	8	-
Special seating	1	1	-
Orthopaedic shoes	1	-	-
Orthotics for cerebral palsy	1	-	-
Orthotics for clubfoot	1	-	-
Ankle-foot-orthotics for human immunodeficiency virus (HIV)	-	1	-
Knee-ankle-foot-orthotics design	1	7	-
**Prosthetic technology**
Upper limb prosthesis	12	4	-
Prosthesis for diabetes patients	2	4	-
Knee-disarticulation prosthesis	-	1	-
Trans-femoral prosthesis	-	1	-
Hip-disarticulation prosthesis	3	5	-
Extension prosthesis for congenital disorders	2	-	-
ICRC polypropylene technology	1	-	-
Advanced technology	4	1	-
Amputations	2	-	-
**Other subjects**
Prosthetic and orthotic services for children	1	1	-
Clinical practice in rural conditions	2	-	-
Biomechanics in depth	6	-	-
Anatomy in depth	2	-	-
Psychology and treating patients with respect	4	-	-
How to access information via the Internet	2	-	-
Workshop management	3	-	-
Basic wheelchair knowledge	1	-	-
Presentation skills to promote the profession	1	-	-
Clinical teamwork	1	-	-
BSc degree programme in prosthetics and orthotics	-	6	-
**Items suggested for removal or reduction from the curricula**
Technical drawing	-	-	8
Metalwork and forging	-	-	8
Woodwork for feet shaping	-	-	7
Reduced mathematics	-	-	3

‘They really need to add spinal orthotics in diploma, we really need to add upper limb prosthesis. We were learning it theoretical is just like do pressure here, do pressure here, and then when you come to the practical doing that pressure is not that easy.’ (Interviewee 11, Tanzania)

#### Drop technical drawing, metalwork and woodwork

Participants perceived that too much time is spent on outdated technology, especially in the first part of the course. Technical drawing, metalwork and woodwork to shape feet were percived as subjects that can be removed from the curriculum. Graduates percived that the amount of time spent on mathematics can be reduced in the curriculum (Table 3).

‘We were shaping feet, but there is no need of shaping feet nowadays. No one ever making feet so with the new technology there is no need of having a lot of shaping feet, because the feet are from industries.’ (Interviewee 17, Tanzania)

### Enabling people to walk is motivating

#### Helping people with disabilities is motivating

Participants described how they liked their profession and how they would like to continue the actual fabrication process of assistive devices. They emphasised that it is motivating to help people walk and to work with rehabilitation when people have disabilities but they are not sick.

‘What I really enjoy is finding that my patients is walking nicely and they are finding a new way in his new life, after being unable to achieve whatever he likes. Patients says that ‘Yes, I can walk. I can do this’, and it’s very good motivation for myself. I become happy, so that is what I really enjoy.’ (Interviewee 17, Tanzania)

#### Low-status profession

Participants perceived they had low salaries and few employment opportunities, which were discouraging aspects of their profession. The profession was described as not well known, having low-status and developing slowly in Africa. Participants described that their work environments were messy, the work was difficult, there were not enough bench workers available and there were no insurance for work injuries.

‘The problem is that our salary is very low and the people are not considering if you are doing a work which is very important. We have many big hospitals but they don’t have these orthopaedic workshops. In our country many people are missing limbs, people have congenital deformities, and lots of other deformities but our government are not considering that.’ (Interviewee 18, Tanzania)

### Obstacles in working conditions

#### Varied support from senior staff and other professionals

Participants described a feeling of confidence when starting to practice what they learnt after graduation and reported good support from senior staff. However, participants also described situations where they were working alone after graduation without the support needed. Medical staff in Tanzania was perceived as having limited knowledge about the prosthetic and orthotic profession. Lack of communication between doctors and prosthetists/orthotists was described as a problem, and increased teamwork was perceived as a possible way to improve services for patients.

‘Whatever I did was considered right and never questioned after graduation; there was no-one who knew better.’ (Interviewee 3, Malawi)

#### Lack of materials

A lack of materials and machines was described by participants as an obstacle to delivering services.

‘There is a lot of time when we don’t have material but a lot of patients, and sometimes we get materials and it takes a lot of time to store. It doesn’t go continually.’ (Interviewee 13, Tanzania)

#### Different demands when working in underserviced and less resourced settings

In rural prosthetic and orthotic workshops, participants described how they have interesting work. They see patients who have been neglected for many years. They often had contractures and described it as difficult to know in what way it was best to start with these patients. Creativity is needed to find good solutions. Patients often do not return as many live far from the service, and assistive devices thus need to be completely finished before they are delivered. Participants describe language barriers to communicating with members of different tribes, and how outreaches to tribes revealed groups of children with disabilities in the bush where there was no housing or access to rehabilitation services. Patients who needed assistive devices could not afford them, or they could not manage or afford to travel very far for the required services. Such situations were perceived as being difficult both in rural and urban settings. The working conditions were perceived as sometimes too difficult in underserviced and less resourced settings, resulting in participants leaving after a while.

‘This is something bad. Some families, when they get a kid who is disabled, the Maasai used to leave them in the house, just hide them, and then you find a kid who has been hiding and now he is no longer a kid. He might be 22 or 23, and he has been in the house for all of his years. He is psychologically defected, and you bring him out and he is just wondering, what is this now, and you see a lot of contractures.’ (Interviewee 11, Tanzania)

### Need for continuous professional development

#### Desire for continued training

Participants described how after graduation they struggled to provide prosthetic and orthotic services to patients with specific diagnoses and therefore there was a desire for further training (Table 2). Participants perceived that patients were sometimes dissatisfied with their work of providing orthoses. Participants also described a desire for further education in the form of a Bachelor of Science degree in prosthetics and orthotics.

‘Spinal orthosis, I mean corset, and I also need to know about upper limb prosthesis, and also special seat orthosis. We need to learn more and recognise those three areas.’ (Interviewee 17, Tanzania)

### Suggested ways of keeping updated

International exchange programmes, the Internet, preparing to teach others, discussions with colleagues and short-term international and national expertise support at centres were described as ways of keeping updated. The ICRC Special Fund for the Disabled short courses in Ethiopia was perceived as a good way for further professional development.

‘I have attended the clinical method of trans-tibial prosthesis in Addis Ababa. That’s why I think I have growing interest in it.’ (Interviewee 1, Tanzania)

## Ethical considerations

Ethical clearance was obtained from the Kilimanjaro Christian Medical College Ethics Committee of Tumani University Makumira (numbers 307 and 274). The participants were informed about the study and written informed consent was obtained. Data were handled confidentially and were stored safely as research data.

## Trustworthiness

To obtain variation of perceptions, participants included both women and men and participants working in two different countries, different sectors and in both rural and city areas. However, the sample only included two African nationalities, which is a limitation of the study. Using three interviewers is a limitation, but the dependability (Lincon & Guba 1985) was strengthened using the same questions, briefing sessions of interviews and the pilot testing of questions. The interviews were conducted in English, which was neither the participants’ nor the interviewers’ first language. This affected the level of communication in a few of the interviews in Tanzania. Credibility was strengthened in the data analysis by the involvement of two of the authors (L.M. and G.A.) in the analysis and the reaching of agreement on descriptive categories and conceptions. Author L.M. is a prosthetist/orthotist with previous research experience in Africa; G.A. is an experienced researcher in qualitative approaches and analysis; and H.G.S. is the dean of TATCOT with long experience within the field of prosthetics and orthotics in Africa. H.G.S. has not been involved in the actual data analysis but contributes to strengthening the credibility of the content of the manuscript (Creswell 2007).

## Discussion

This study contributes to the description of possible areas for improvement or revision of the current education programme at TATCOT and the international guidelines for prosthetic and orthotic education. The main findings show that there is a need for revision of the curriculum of the diploma course at TATCOT. The diploma course is a Category II course according to the guidelines for training personnel in developing countries in prosthetics and orthotics service (World Health Organization & International Society of Prosthetics and Orthotics 2005).

The major changes to the curriculum suggested were to reduce the time spent on learning to work with metal and wood and to focus more on advanced prosthetic and orthotic technology. This confirms similar results obtained in a previous survey of TATCOT graduates in 2006 (Jacobs 2007). The revised curricula implemented in 2011 at TATCOT have reduced time spent on metalwork. Woodwork related to producing components has been omitted (Tanzanian Training Centre for Orthopeadic Technologists 2010). TATCOT would like to provide modern training that adapts to developments in different countries (Raab 1992). The technology used in low-income countries has changed and, at least in Malawi and Tanzania, is low-cost technology, that is polypropylene technology developed by the ICRC is commonly used (Sexton *et al*. 2012). This development is dependent on the provision of components by the ICRC or similar provisions to Africa. However, it is also to be considered that it is not in every setting that graduates will be able to obtain off-the-shelf components like the ICRC components. Skills of producing components for the prosthetic/orthotic devices are needed for people practicing in low-income countries. It should also be noted that students at a training institution need to be exposed to different technologies and not just one technology. Sustainability needs to be carefully considered when it comes to recommending changes to the curriculum.

In order to implement the CRPD, the quality of prosthetic and orthotic services in low-income countries is important (United Nations 2007). Previous studies indicated that the majority of patients used their prosthetic or orthotic devices (90% in Malawi and 86% in Sierra Leone) produced mainly with ICRC polypropylene technology. However, patients’ self-reported mobility and satisfaction of assistive device revealed that the design and manufacture of prostheses and orthoses using low-cost technology needs be improved. A focus on improvements in-service delivery needs to be directed towards increasing the ability of patients to ambulate on uneven surfaces, hills and stairs, as well as increasing patients’ ability to walk long distances with reduced pain (Magnusson *et al*. 2013, 2014). This suggests that the ICRC polypropylene technology needs to improve or be replaced by better solutions. A survey answered by the staff providing prosthetic and orthotic services from a number of low-income countries indicated that the development of improved designs for durable and low-cost components, in particular prosthetic knees and feet, needed to be addressed. Poor alignment of prosthetic and orthotic devices was also widely reported (Wyss *et al*. 2015).

The results illustrate that the graduates struggled to provide assistive devices such as spinal orthotics and upper limb prosthetics, as they had only a basic theoretical education of such devices. Similar results were obtained in a previous survey at TATCOT (Jacobs 2007) and a later impact assessment also indicates a need to cover upper limb prosthetics within the education (Sexton *et al*. 2012). The results (Table 2) indicate that biomechanics in depth needs to be added in the curricula; this was also confirmed in the impact assessment report (Sexton *et al*. 2012).

In 1992, a survey found that too few professionals were being educated and that most of the workforce providing services had not gone through an education programme (Shangali 2002). It is thus important that prosthetists/orthotists completing an education programme can assist not just with the most common and basic groups of patients in need of assistive devices but also patients with less common difficulties as not everyone will have the opportunity to receive further training. The graduates perceived that they were not prepared for underserviced and less resourced settings, working conditions or providing services to patients who had been without services for years. If the CRPD (United Nations 2007) is to be implemented, prosthetic and orthotic services need to be available beyond a few rehabilitation centres in the biggest cities in Africa. To implement the CRPD, prosthetic and orthotic services need to be scaled up in low-income countries and provided to all persons with disabilities who could potentially benefit from prosthetic and orthotic devices (Magnusson 2014). In order to scale up services, increased numbers of African prosthetists/orthotists are needed. This is an urgent issue, not least in light of the fact that many today have not yet received any services at all. However, it is a positive development that a number of new prosthetic and orthotic education programmes have started in recent years in Africa.

To provide a good learning context, it was emphasised that equal treatment by teachers and staff regardless of the ethnic background or gender of the student was important. Unequal treatment related to ethnic background was reported, but women were treated well by teachers at TATCOT, in contrast with results obtained in a previous study for the Pakistan Institute for Prosthetic and Orthotics Service (PIPOS) (Magnusson & Ramstrand 2009).

The findings both of the present study and of previous studies concerning Sierra Leone (Magnusson & Ahlström 2012) and Pakistan (Magnusson & Ramstrand 2009) demonstrate that managing specific pathological conditions and problems with materials are common difficulties perceived by graduates from both TATCOT and PIPOS. In the revised curricula implemented 2011, assessments of patients have been introduced earlier in the education at TATCOT (Tanzanian Training Centre for Orthopeadic Technologists 2010). A need for further education and a desire for continuous professional education are also common themes. TATCOT and PIPOS graduates desired and perceived a need for improvements in the education programmes. They perceived that more time needed to be dedicated to applied biomechanics and the clinical management of various specific orthotic and prosthetic patient groups, such as cerebral palsy patients, diabetic patients, arm amputees and patients in need of spinal orthotics. They also perceived a need to include education related to advanced prosthetic and orthotic technology. Our findings confirm previous results for graduates of TATCOT and PIPOS (Magnusson & Ramstrand 2009) that suggested revisions of curricula including reduced technical drawing, metalwork and mathematics. The study in Pakistan found that the curriculum needed to add psychology, which in this study emerged only in few interview statements probably because applied psychology and reflective clinical practice have been taught at TATCOT. A teaching module delivered at TATCOT aims at providing the students with the ability to move from a practising orthotist/prosthetist to one of a reflective practitioner (Grobler, Van Schalkwyk & Wagner 2006). Additionally, the graduates from both TATCOT and PIPOS desired teachers with a higher degree and they experienced good support from senior staff when entering the workforce.

## Practical implications

As TATCOT is following current international guidelines, this study contributes to the review and update that was planned by ISPO/WHO of the international guidelines for training personnel in Africa, Asia and South America for prosthetics and orthotics services. The current international guidelines for Category II, prosthetist/orthotist is used by all the ISPO-certified prosthetic and orthotic education programmes in these three continents (World Health Organization & International Society of Prosthetics and Orthotics 2005).

Locally, this study suggests that there is a need to improve information about the profession to students before they start the education. It also suggests that teaching methodology and inequality in the treatment of students need to be addressed. The study further suggests that there is a need for a number of changes in the curriculum such as increasing the attention devoted to clinical management of different groups of patients and applied biomechanics, with less focus on technical drawing, metalwork and woodwork. Also extra support or more resources need to be provided for graduates working in under-resourced/rural settings. The results also indicate that there is a need to give prosthetists/orthotists the opportunity to attend formal and short-term tailored courses on specific specialities, i.e. upper limb orthotics/prosthetics, spinal orthotics, biomechanics and different designs of lower limb prosthetics/orthotics. This could be through a structured course within a country/region where a large number of participants can attend. It is also suggested that there should be in-service training courses within their individual facilities.

## Conclusion

There was low awareness of the profession in general and amongst students entering the education programme. Enabling people to walk was a motivating factor of the profession whilst the low salary was a discouraging factor. The learning environment was perceived as good with good teachers and well-equipped teaching facilities. To improve the learning environment, issues of equality for students from different countries should be addressed. Improvements in the pedagogical approach and higher competence level of teachers are needed to improve the education provided at TATCOT and to provide a better service to patients. When entering the workforce, graduates perceived that they lacked knowledge in dealing with specific clinical problems, were not prepared for working in rural conditions and they desired continued training. Therefore, more time in education should be dedicated to the clinical management of different groups of patients and applied biomechanics and focus on technical production aspects of prosthetic and orthotic practice should be less.
